# Institutional Notes and News

**Published:** 1910-01-29

**Authors:** 


### Institutional Notes and News.

**New Extensions and Needs.**

Reference was made in the report at the quarterly meeting of the Governors of the Leicester Infirmary to the opening of the new nurses' home by Mrs. Fielding Johnson (the wife of the ex-chairman of the Board) at the beginning of February. Alluding to the needs of 1910, it was stated there was urgent need for £3,000 per annum additional income to maintain 33 additional beds to be used on the occupation of the nurses' home and for the increased cost of maintenance of the new nurses' home. The £400 set aside to the building up of a reserve fund would be required in the course of the year for : (1) The conversion of the present nurses' sitting room and the enlargement of the present dining room. (2) The improvement of the mortuary, which, although not inefficient, needed bringing up to the higher standard of ideas as compared with ten years ago. (3) The painting of the exterior of the south-east wing, which would probably be undertaken. On the reconstruction scheme Sir Edward Wood reported that £100,000 had been expended, and he appealed for contributions in liquidation of the balance of £3,000 still to be raised. The report concluded with a cordial acknowledgment of the work of the honorary medical and surgical staff, of Miss Rogers (the lady superintendent) and the nursing staff for their devotion to the interests of the patients, and to the committee of the Saturday Hospital Society, and Mr. Woolley, their secretary, for the achievement of the new record of £12,850.

**Norfolk and Norwich Hospital**

At the last quarterly meeting of the governors of the Norfolk and Norwich Hospital, the Chairman said the past quarter would be always memorable in the history of the hospital, first on account of the visit of the King on October 25, and secondly, on account of the laying of the foundation-stone of the new buildings, which marked the day of the extending usefulness of the hospital. The report stated that the work on the septic and isolation block would be begun immediately, such delay as had been caused was due to the intricacy of the building, which had meant more time in its specification than had been expected. Archdeacon Pelham, in seconding the adoption of the report, said that the ordinary receipts by no means kept pace with the expenditure, but it would be a great pity when they were trying to raise a large sum for additional buildings if they made too much of the present position.

**Deficit at Lincoln Hospital.**

The yearly financial statement of the Lincoln County Hospital was made at the quarterly meeting on January 13. It showed that in spite of the increase of £155 on the receipts, from various sources there was a deficit on the year of £825. Receipts totalled £6,468, and expenditure £7,293. The secretary further pointed out that the tradesmen's bills had been £3,787—in other words, had increased by £1,300. That, he went on, was a very large increase, and they must remember that in 1908 these bills had already gone up by £232, making a total increase on the two years of £1,500. He was afraid that the £500 they had transferred to the capital account would have to be withdrawn and placed again to the income account. The chairman, remarking that the expenditure on the fabric had been very large during the year, said he had fancied it might be worse, and the accounts were thereupon passed without comment.

**Votes of Condolence.**

The Dewsbury and District Infirmary Board, at its monthly meeting on Tuesday, had the melancholy duty of passing votes of condolence with the relatives of three prominent local gentlemen who have recently passed away, namely, Dr. Broughton, Sir T. F. Firth, and Major Chaley Fox. Dr. Broughton was a member of the honorary medical staff for about thirty years. At one time in the history of the institution there was a divergence of opinion between the staff and the board, which led to the dislocation, for the time being, of the staff. Rather than let the institution suffer, however, for lack of medical advice and attention Dr. Broughton came to the rescue. Major Fox had not only the good of that institution at heart, but he had the interests at heart of every institution that was for the good of the people of this district. He was a liberal supporter of the infirmary and organised many successful bazaars in its aid. Sir T. F. Firth had done a tremendous amount of good for the institution in years gone by, and his firm had for many years subscribed a sum equal to that which was collected by the workpeople. The resolution in each case was carried by the members rising in their places.

**Plymouth Royal Eye Infirmary.**

The Royal Eye Infirmary, Plymouth, held its annual meeting on January 19, at which it was announced that the in-patients had increased from 278 to 300, 39 of whom had contributed at the rate of half a sovereign a week. The expenditure was less than the receipts, which amounted to £1,845, of which £115 had been contributed by the out-patients. The Mayor, who presided, said that so high was the hospital's status that it was a question whether Plymouth or London stood first in the matter of skill for ophthalmic patients. Mr. Charles King was elected chairman for the current year, and Mr. Cyril Pode suggested that the hospital's success warranted the accumulation of a reserve fund against the day when their buildings would need enlargement.

**Leicester Infirmary.**

The interesting and satisfactory annual report of the Leicester Infirmary, which was presented at the quarterly meeting of governors on January 19, will come as no surprise to readers of The Hospital, who will remember our report on this institution in the issue of December 11. Sir Edward Wood, whose work for the infirmary has made him famous, presided. The income last year was sufficient to meet the deficits of previous years, to pay the annual expenses, and even to leave a small balance in hand. The Hospital Sunday Fund, legacies, and an increase in the value of the invested funds of the hospital were the main factors in this success. The report went on to state that the 33 new beds in the new ward would be available shortly. If last year's income is maintained the cost of the upkeep of this ward should be met out of it. A sum of £100 to £150 is needed to bring the mortuary up to date, a need which was emphasised in The Hospital's report already quoted. Sir Edward Wood moved the adoption of the report, and said it was a credit to the industrial classes that they provided nearly one-half of the income. He pointed out the need for a further £3,000 a year to maintain the additional accommodation and the new nurses' home without debt.

